# The Genome of the North American Brown Bear or Grizzly: *Ursus arctos* ssp. *horribilis*

**DOI:** 10.3390/genes9120598

**Published:** 2018-11-30

**Authors:** Gregory A. Taylor, Heather Kirk, Lauren Coombe, Shaun D. Jackman, Justin Chu, Kane Tse, Dean Cheng, Eric Chuah, Pawan Pandoh, Rebecca Carlsen, Yongjun Zhao, Andrew J. Mungall, Richard Moore, Inanc Birol, Maria Franke, Marco A. Marra, Christopher Dutton, Steven J. M. Jones

**Affiliations:** 1 Canada’s Michael Smith Genome Sciences Centre, British Columbia Cancer Agency, Vancouver, BC V5Z-4S6, Canada; hkirk@bcgsc.ca (H.K.); lcoombe@bcgsc.ca (L.C.); sjackman@bcgsc.ca (S.D.J.); cjustin@bcgsc.ca (J.C.); ktse@bcgsc.ca (K.T.); dcheng@bcgsc.ca (D.C.); echuah@bcgsc.ca (E.C.); ppandoh@bcgsc.ca (P.P.); rthorne@bcgsc.ca (R.C.); yzhao@bcgsc.ca (Y.Z.); amungall@bcgsc.ca (A.J.M.); rmoore@bcgsc.ca (R.M.); ibirol@bcgsc.ca (I.B.); mmarra@bcgsc.ca (M.A.M.); sjones@bcgsc.ca (S.J.M.J.); 2 Department of Medical Genetics, University of British Columbia, Vancouver, BC V6T-1Z4, Canada; 3 Conservation and Wildlife Department, Toronto Zoo, Toronto, ON M1B-5K7, Canada; mfranke@torontozoo.ca (M.F.); cdutton@torontozoo.ca (C.D.); 4 Department of Molecular Biology and Biochemistry, Simon Fraser University, Burnaby, BC V5A-1S6, Canada

**Keywords:** grizzly bear, *Ursus arctos* ssp. *horribilis*, genome, microfluidic partitioning, nanopore

## Abstract

The grizzly bear (*Ursus arctos* ssp. *horribilis*) represents the largest population of brown bears in North America. Its genome was sequenced using a microfluidic partitioning library construction technique, and these data were supplemented with sequencing from a nanopore-based long read platform. The final assembly was 2.33 Gb with a scaffold N50 of 36.7 Mb, and the genome is of comparable size to that of its close relative the polar bear (2.30 Gb). An analysis using 4104 highly conserved mammalian genes indicated that 96.1% were found to be complete within the assembly. An automated annotation of the genome identified 19,848 protein coding genes. Our study shows that the combination of the two sequencing modalities that we used is sufficient for the construction of highly contiguous reference quality mammalian genomes. The assembled genome sequence and the supporting raw sequence reads are available from the NCBI (National Center for Biotechnology Information) under the bioproject identifier PRJNA493656, and the assembly described in this paper is version QXTK01000000.

## 1. Introduction

The grizzly bear is the most common sub-species of brown bears found in North America. Brown bears (*Ursus arctos*) were historically found across much of North America, Asia, Europe, and even Northern Africa, but loss of habitat, human encroachment, and hunting have seen this range greatly reduced in the past two centuries [1]. Within North America, both the California Grizzly *(Ursus arctos californicus*) and the Mexican Grizzly (*Ursus arctos nelsoni*) are already extinct. The North American brown bear is among the largest predators on the continent, second only to its close relative the polar bear and, as such, requires a large territory to sustain its diet. Grizzly bear density varies from 3 per 100 km^2^ in the resource scarce interior of British Columbia, to as many as 25 per 100 km^2^ in resource dense coastal regions [2]. As human encroachment on grizzly habitat continues, a better understanding of their biology will aid in conservation.

DNA was sequenced from a 20-year old male grizzly bear, Samson. This bear was orphaned in the wilds of Alaska in 1998 when he was less than a year old. He was rescued and raised at the Alaska Children’s Zoo and then moved to the Toronto Zoo.

## 2. Methods

The genome assembly was constructed from both an Illumina (San Diego, CA, USA) HiSeqX sequenced Chromium library and an Oxford Nanopore (Oxford, UK) library sequenced on a MinION sequencer. The raw sequence data came completely from the Chromium library; the nanopore reads were used only for scaffolding information to increase the assembly contiguity. 

A blood sample was taken as part of a routine physical exam of an adult male North American brown bear at the Toronto Zoo (GAN/ISIS:MIG12-29695490/34125). The portion of the sample used for sequencing was deemed to be in excess after the animal’s physical health was ascertained and was donated by the zoo. The Toronto Zoo operates under the accreditation of AZA (Accredited Zoos and Aquariums), CAZA (Canada’s Accredited Zoos and Aquariums), and all of their research is carried out under a certification of Good Animal Practice^®^ awarded by CCAC (Canadian Council on Animal Care).

High molecular weight (HMW) DNA was extracted from a fresh whole blood sample using the Qiagen MagAttract HMW DNA Kit (cat. no. 67563, QIAGEN, Germantown, MD, USA) and the HMW genomic DNA (gDNA) extraction protocol as detailed in the Chromium Genome Reagent Kits Version 2 User Guide (PN-120229) [3,4]. Integrity of the DNA was assessed by Pulsed Field Gel Electrophoresis (PFGE) with the majority of DNA fragments over 50 kb in length. The fragment size was confirmed in silico after assembly; the weighted mean molecule length was 46 kb.

A micro-fluidic partitioned library was created using the Chromium system from 10× Genomics (10× Genomics, Pleasanton, CA, USA). GEMs (Gel beads-in-EMulsion) were produced by combining DNA, Master Mix, and partitioning oil in the 10× Genomics Chromium Controller instrument with the micro-fluidic Genome Chip (PN-120216) (10× Genomics). The DNA in each GEM underwent isothermic amplification as a barcode was added to each fragment. Barcoded fragments then underwent Illumina library construction (as per the Chromium Genome Reagent Kits Version 2 User Guide (PN-120229)).

The resulting library was assessed for quality using the Agilent 2100 Bioanalyzer (Santa Clara, CA, USA) and a DNA 1000 assay. The median insert size was 375 bp. The library was then sequenced on an Illumina HiSeqX sequencer using the paired-end protocol to produce 855 million 150 bp reads, an estimated 55-fold genome coverage.

A second genomic library was constructed from the same HMW DNA sample, but this one conformed to Oxford Nanopore Technologies’ protocols. It was constructed using the SQK-LSK108 Ligation Library Kit. Liquid handling was performed using wide bore tips to avoid physically breaking the DNA. Six µg of HMW DNA were gently sheared using 10 passes up and down through a 26 gauge needle (BD medical, Franklin Lakes, NJ, USA, cat. no. 309625) and a size selection step was completed using a 0.35:1 ratio of PCRClean DX magnetic beads to DNA (cat. no. C-1003-450, ALINE Biosciences, Woburn, MA, USA). NEB Ultra II (New England Biolabs, Ipswich, MA, USA, cat. no. E7646A) was used for end-repair and 3′ A-tailing. NEB Blunt/TA Ligation Master Mix (M0367S) was used to ligate the Oxford Nanopore adapters. A final size selection of 0.4:1 ratio (magnetic beads to library) was done to eliminate smaller molecules. MinION sequencing proceeded using the FLO-MIN106 (R9 Version) flow cell and the software programs MinKnow 1.13.1 and GUI 2.0.13. The MinION sequencing run produced over 1 million reads, totaling 16 billion base pairs, with an N50 of 20,211 bp. This amount of data equates to a 7-fold genome coverage.

Supernova (version 2.0.1) [5] was used to assemble the Chromium Illumina reads. The 855 million reads (55× coverage) were assembled into an initial assembly of 8474 scaffolds, totaling 2.31 Gb in length and an N50 of 33.78 Mb. Tigmint [6] was used to break potential misassemblies, based on read coverage across the assembly. This provided a starting point for re-scaffolding that was as error free as possible. ARCS [7] was run to scaffold the contigs using the Chromium reads, thereby making the most of this data. LINKS [8] was then used to add scaffolding information to the assembly from the Nanopore reads. LINKS is a tool designed to use uncorrected long reads for scaffolding and does not introduce any new bases to the assembly. The tool was run iteratively eight times, each time using a larger fragment size to produce pseudo read-pairs from the Nanopore data that were then applied to the assembly as scaffolding. Within LINKS, the distance values of 1000, 2500, 5000, 7500, 10,000, 12,500, 15,000, and 30,000 were used successively to achieve the scaffolding results observed in Table 1.

Finally, Sealer [9] was used to fill in the gaps that the previous scaffolding steps had produced. Because the Illumina reads from the Chromium library are substantially more accurate than the Nanopore reads, the Bloom filter for the Sealer run was populated using only the former read set.

At each step of the assembly, BUSCO [10] was used as an additional measure to assess genome completeness. BUSCO (Benchmarking Universal Single-Copy Orthologs) attempts to reconstruct a set of 4104 conserved mammalian genes, and the number of genes reconstructed is an indicator of genome assembly completeness. Ninety-six point one percent of the BUSCO mammalian gene set was reconstructed as complete genes in this assembly, and a further 1.9% of the genes were identified as gene fragments. The high reconstruction rate of BUSCO genes was constant at all stages of the assembly process, indicating both the high quality of the assembly and that gene rich regions are more likely to be assembled correctly from the start due to their high information content. BUSCO genes represented approximately 20% of all grizzly bear genes, and extrapolations from this data set are premised upon these genes being representative of all genes in both their distribution and structure.

The genome was annotated using the RefSeq eukaryotic pipeline [11]. This analysis indicated the presence of 19,848 coding genes, 7061 non-coding genes, 3671 pseudo-genes, and 119 immunoglobulin gene segments.

## 3. Results and Discussion

The grizzly bear genome has a diploid karyotype of 37 chromosome pairs [12,13], and there is a mean distance of 688 bp between heterozygous positions in this assembly. Based on the N50 of our assembly and the estimated genome size of 2.3 Gb, the longest scaffolds in the grizzly bear assembly most likely represent full chromosome arms, and the observed heterozygous positions can act as a starting point for further population diversity studies.

The polar bear is the closest relative to the grizzly bear for which the genome has been sequenced [14]. Based on BUSCO analysis of both assemblies, using the 4301 gene mammalian dataset, the grizzly bear genome is more complete. The grizzly bear genome is also more contiguous than the polar bear genome as detailed in Table 2.

**Figure 1:**
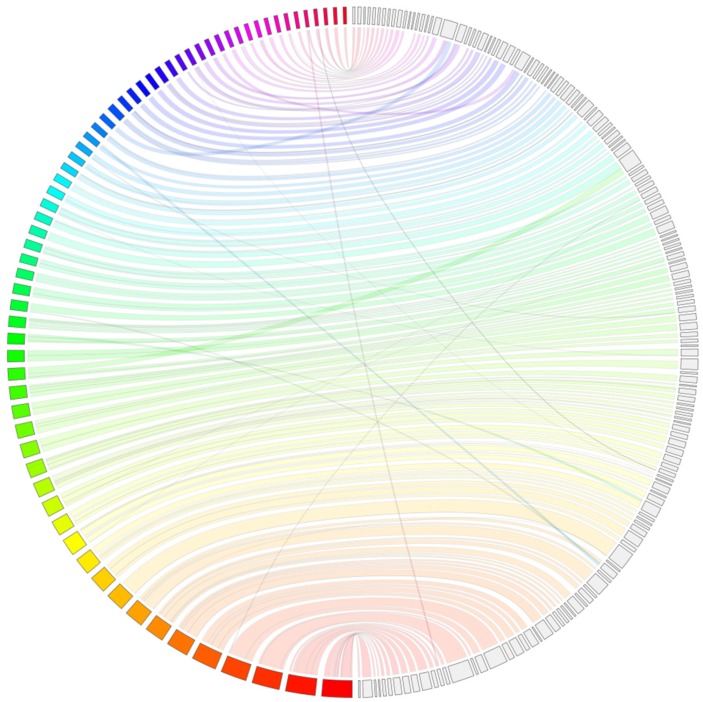
Jupiter plot. A global genome alignment, using BWA-MEM, of the grizzly genome (left side of circle) to the polar bear genome (right side). Connections show the aligned regions of each assembly. The grizzly scaffolds are limited to those over 10 Mb in length (>85% of the assembly). The longest polar bear scaffolds were selected to sum to the same amount of sequence (2 Gb). Only alignments over 10 kb in length are displayed.

A global alignment of the grizzly bear assembly to the polar bear assembly was accomplished using BWA-MEM (version 0.7.17) [15] and visualized as a Jupiter plot [16] (an adaptation of a Circos diagram [17]) in Figure 1. The colored bands of Figure 1 represent regions of synteny. Diagonal lines in the plot identify split alignments that may be the result of rearrangements, but more likely these diagonal lines point to differing break-points in contiguity between the two assemblies, since they all appear on the edges of scaffolds. At 10 kb resolution, there were no definite breaks in synteny between the two assemblies.

Micro-fluidics has proven to be a reliable technology for creating partitioned libraries. These libraries, in turn, greatly enhance the quality of the resulting genome assembly. Nanopore reads provide a great deal of scaffolding information, from an independent source, that can corroborate the integrity of a Supernova assembly. This combined approach to genome assembly has produced a high-quality reference genome. A reference brown bear genome can serve as a solid starting point for further investigation of the evolutionary ties to other bear species and the intra-species diversity present within the grizzly bear population.

## Figures and Tables

**Table 1:** Assembly statistics and gene content for the genome sequences reported in this study. Busco: Benchmarking Universal Single-Copy Orthologs.

Assembly	# of Scaffolds	Gaps within Scaffolds	Scaffold N50 (bp)	Longest Scaffold (bp)	BUSCO Complete Genes (of 4104)
Supernova	8474	21,957	33.78 × 10^6^	105.9 × 10^6^	3943 (96.1%)
Tigmint	8728	21,947	26.32 × 10^6^	92.41 × 10^6^	3943 (96.1%)
ARCS	8679	21,996	27.77 × 10^6^	92.41 × 10^6^	3943 (96.1%)
LINKS1	8350	22,219	27.77 × 10^6^	92.41 × 10^6^	3943 (96.1%)
LINKS8	6673	23,947	36.71 × 10^6^	92.42 × 10^6^	3943 (96.1%)
Sealer	6673	15,572	36.71 × 10^6^	92.43 × 10^6^	3943 (96.1%)

**Table 2:** Assembly statistics of the grizzly bear and its closest sequenced relative, the polar bear.

Assembly	# of Scaffolds	Scaffold N50 (bp)	Scaffold L50	# of Contigs	Contig N50 (bp)	Contig L50	BUSCO Complete Genes
Grizzly Bear	6673	36.71 × 10^6^	21	22,245	314 × 10^3^	2191	3943 (96.1%)
Polar Bear	23,819	15.94 × 10^6^	46	134,162	46 × 10^3^	14,124	3890 (94.7%)
